# Artificial light impairs local attraction to females in male glow-worms

**DOI:** 10.1242/jeb.245760

**Published:** 2023-06-14

**Authors:** Estelle M. Moubarak, A. Sofia David Fernandes, Alan J. A. Stewart, Jeremy E. Niven

**Affiliations:** School of Life Sciences, University of Sussex, Falmer, Brighton BN1 9QG, UK

**Keywords:** Artificial lighting at night, ALAN, Mate attraction, Mate choice, Light pollution, Lampyridae, Y-maze

## Abstract

The negative effects of artificial lighting at night (ALAN) on insects are increasingly recognised and have been postulated as one possible cause of declines in insect populations. Yet, the behavioural mechanisms underpinning ALAN effects on insects remain unclear. ALAN interferes with the bioluminescent signal female glow-worms use to attract males, disrupting reproduction. To determine the behavioural mechanisms that underpin this effect of ALAN, we quantified the effect of white illumination on males' ability to reach a female-mimicking LED within a Y-maze. We show that as the intensity of illumination increases, the proportion of males reaching the female-mimicking LED declines. Brighter illumination also increases the time taken by males to reach the female-mimicking LED. This is a consequence of males spending more time: (i) in the central arm of the Y-maze; and (ii) with their head retracted beneath their head shield. These effects reverse rapidly when illumination is removed, suggesting that male glow-worms are averse to white light. Our results show that ALAN not only prevents male glow-worms from reaching females, but also increases the time they take to reach females and the time they spend avoiding exposure to light. This demonstrates that the impacts of ALAN on male glow-worms extend beyond those previously observed in field experiments, and raises the possibility that ALAN has similar behavioural impacts on other insect species that remain undetected in field experiments.

## Teaser

**Highlighted Article:** Under brighter illumination, fewer glow-worm males reach a female-mimicking LED in a Y-maze, taking longer to do so, partly because they retract their head beneath their head-shield for longer.

## INTRODUCTION

The presence of artificial lighting in the environment at night is increasingly recognised to have deleterious effects on many aspects of behaviour and ecology in arthropods and vertebrates including humans (Gaston et al., 2015; Hölker et al., 2010; Longcore and Rich, 2004; Royal Commission on Environmental Pollution, 2009). Artificial lighting at night (ALAN) may be a particularly potent form of environmental pollution because it is both widespread (e.g. Falchi et al., 2016) and patterns of daylight and darkness have been fixed and predictable over long periods as they are determined by the orbit of the Earth around the Sun and the Moon around the Earth. Indeed, ALAN has been implicated in large-scale insect declines that threaten the functioning of ecosystems and the provision of ecosystem services (Grubisic et al., 2018; Owens and Lewis, 2018).

ALAN has been shown to affect a variety of behavioural and ecological processes in insects (e.g. Davies et al., 2013; Gaston et al., 2015; Owens and Lewis, 2018; Boyes et al., 2021). Perhaps the most well-documented effects are those on lepidopterans, particularly moths, showing that ALAN disrupts multiple aspects of their biology across different life history stages (e.g. Macgregor et al., 2017; Van Grunsven et al., 2020; Boyes et al., 2021). The effects of ALAN have been documented on pollination (e.g. Knop et al., 2017; Macgregor et al., 2017; Giavi et al., 2021) as well as intraspecific communication and reproduction (e.g. Elgert et al., 2020; Stewart et al., 2020; Van den Broeck et al., 2021; Kivelä et al., 2023). ALAN can also disrupt interactions between insect species at different trophic levels (e.g. Davies et al., 2013; Sanders et al., 2018). Yet the mechanisms by which ALAN affects various aspects of insect biology remain unclear for most species in terms of both a mechanistic understanding of their behaviour and their underlying physiology (Gaston et al., 2013, 2015; Kalinkat et al., 2021).

Adult common glow-worms (*Lampyris noctiluca*) engage in nocturnal mating behaviour during which females use a bioluminescent signal, the ‘glow’, to attract males (Tyler, 2002). The larviform wingless females move little as adults, with adult dispersal dependent on the volant males (Tyler, 2002). Males detect glowing females using photoreceptors sensitive to the glow in their large superposition compound eyes and move towards them (Booth et al., 2004). It is this reliance on a visual signal that makes glow-worms particularly susceptible to the effects of ALAN. Indeed, several studies have posited that recent population and range declines in glow-worms are caused, at least in part, by ALAN (e.g. Gardiner, 2007; Gardiner and Tyler, 2002; Owens and Lewis, 2018; Gardiner and Didham, 2020, 2021), though the evidence is largely correlative.

ALAN has the potential to reduce males' ability to reach females or LEDs mimicking the female glow (e.g. Ineichen and Rüttimann, 2012; Bird and Parker, 2014; Elgert et al., 2020; Stewart et al., 2020; Van den Broeck et al., 2021; Kivelä et al., 2023), which may ultimately affect the viability of glow-worm populations by reducing their reproductive potential. Experimental evidence for the role of ALAN on mating in common glow-worms comes primarily from experiments in which artificial lighting is introduced into sites that contain females but ordinarily lack direct illumination (e.g. Bird and Parker, 2014; Stewart et al., 2020; Van den Broeck et al., 2021; Kivelä et al., 2023). Male glow-worms are trapped using a green LED to mimic the female glow (a dummy female), allowing the effects of direct illumination to be quantified in terms of males captured (e.g. Bird and Parker, 2014; Stewart et al., 2020; Van den Broeck et al., 2021; Kivelä et al., 2023). In at least one case, however, females rather than LEDs have been experimentally exposed to direct illumination showing that they fail to move away from artificial light sources (Elgert et al., 2020). These studies demonstrate that even low intensities or specific wavelengths of artificial lighting reduce the numbers of males able to reach females or female-mimicking LEDs with potentially deleterious effects on glow-worm populations.

The experiments described above demonstrate the effects of artificial lighting on the glow-worm signalling system in the field, but quantification and mechanistic insight are lacking. There are many interpretations of the reduction of males attracted to illuminated females in field experiments. For example, directly illuminated females may be detected by males but perceived as lower quality (Hopkins et al., 2012) so that males seek alternatives. Alternatively, males may still be attracted to directly illuminated females but may themselves be affected by exposure to artificial light, becoming disorientated or unable to move. To complement existing field studies, we quantified the effect of white illumination on the ability of males to reach female-mimicking LEDs within a Y-maze. Here, we show that increasing the intensity of white illumination prevents greater numbers of males from reaching the female-mimicking LED and increases the time males take to reach them. This is a consequence of males spending more time in the central arm of the Y-maze and with their head retracted beneath their head shield, possibly to attenuate the light to which the compound eye is exposed. However, the time males spend in the vicinity of the female-mimicking LED once reached is unaffected by the intensity of illumination. We also show that males exhibit a pronounced decline in mate attraction upon successive exposure to females.

## MATERIALS AND METHODS

### Animals

Male glow-worms [*Lampyris noctiluca* (Linnaeus 1767)] were collected using custom-made lures consisting of a green LED (555 nm; SSL-LX5093PGD, Lumex Inc., Carol Stream, IL, USA) mounted above a funnel trap (Booth et al., 2004). Between 2 and 15 males were maintained in clear Perspex boxes (22×13×14 or 17×11×5 cm, length×width×height) at room temperature (21–23°C) in high-humidity conditions with a horizontal black platform beneath which they could shelter. The boxes were illuminated by white light (100 lx) produced by LED strips (ATOM LED lighting, Telford, UK) plugged into programmable mechanical timer switches, following a 10 h:14 h dark:light cycle. Glow-worms were maintained in this diel rhythm for at least 48 h prior to an experiment. Twenty-four hours prior to each experiment, males were individually marked with acrylic paint and had a small pin attached to their pronotum with UV-activated liquid plastic bonding filler (VR7, Edinburgh, UK). The pin allowed easy placement of the males in and out of the Y-maze, and prevented them from flying. The pin could be removed after the trials were complete without apparent harm to the males.

### Attraction experiments

**Fig. 1.**
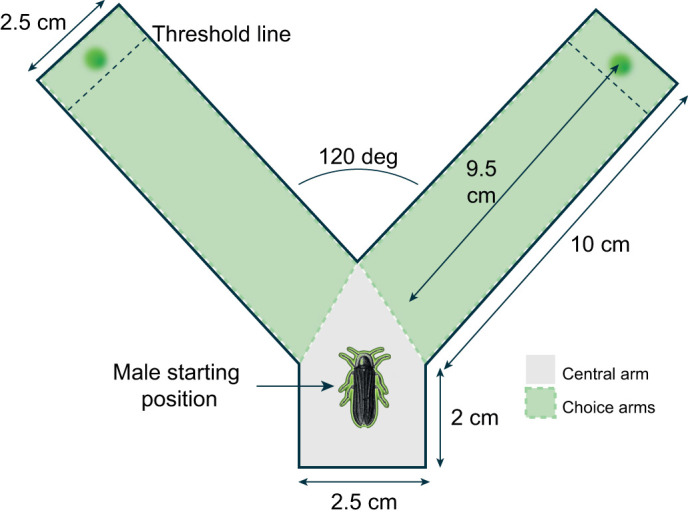
**A schematic of the Y-maze.** The Y-maze is shown from above with indications of the males' starting position in the central arm, the position of the LED in each choice arm, and the dimensions (cm) of key features. The threshold line indicates the region 1 cm from the distal end of the Y-maze.

We constructed a bespoke Y-maze with a short central arm (2×2.5 cm, length×width) and two choice arms (10×2.5 cm, length×width) separated by a 120 deg angle (Fig. 1). A single green LED with a narrow emission spectrum (555 nm; SSL-LX5093PGD, Lumex; Fig. S1A) was mounted 0.5 cm from the end of each choice arm. Males were allowed to explore the Y-maze for 5 min before the start of the experiment. Then, trials began with the male placed in the central arm so that both choice arms were visible. An LED was turned on in one choice arm at random; the intensity from the male starting location was 1 lx. Each trial lasted 5 min, after which the LED was turned off, the glow-worm returned to the starting position and the LED in the opposite arm turned on. Each experiment consisted of six sequential trials executed in a Y-maze unlit other than the green LED or illuminated with diffuse ‘warm’ white light (KL1500 LCD, Schott, Mainz, Germany; Fig. S1B) from 25 cm above with increasing intensity (25, 45, 90 and 145 lx) during the second to fifth trials. In some trials the white light was focused on the final 2.5 cm of each choice arm of the Y-maze, though there was no significant difference between the results of these trials and those with diffuse light (e.g. Fig. S2). Each set of six trials involved a different male glow-worm.

### Video capture and analysis

The Y-maze was illuminated from below with diffuse infrared lighting (>690 nm), which is not detected visually by most insects (for review, see Goldsmith and Bernard, 1974). Videos of the Y-maze were captured at 20 frames s^−1^ using a USB infrared camera (ELP-USBFHD05MT-RL36-U, Shenzhen Ailipu Technology Co., China). Video recordings of individual trials were analysed offline using ImageJ (Schindelin et al., 2012). The location of the glow-worm (which appeared black on the white background) was identified by measuring the minimum pixel brightness for an RGB image in regions of interest covering the central arm, the first 5 cm of each long arm, the next 5 to 8.5 cm of each long arm, and the 1.5 cm surrounding the LED.

### Spectrometry and light intensity

We used a commercial spectrometer (CCS100/M, Thorlabs, Newton, NJ, USA) to measure the emission spectra of the green LED and the white light source used to illuminate the Y-maze (Fig. S1). Spectra were recorded using OSA software (Thorlabs). The intensity of the light sources was measured using a commercial photometer (ALX-8809A, ATP Instrumentation, Ashby-de-la-Zouch, UK).

### Statistical analysis

All statistical analyses were conducted in R 4.0.3 (https://www.r-project.org/). The numbers of males reaching LEDs were analysed in both the control (dark Y-maze) and treatment (illuminated Y-maze) using binomial family generalised linear models (GLMs) from the ‘stats’ package, allowing count data as a response. Pairwise differences in the number of glow-worms successfully reaching the LED between the control and the light treatment were assessed using a *G*-test from the ‘descTools’ package (https://cran.r-project.org/package=DescTools). A log-linear Gaussian regression from the ‘stats’ package was used to model the time taken by males in several behaviours (time taken to reach luminous LED, time spent near the luminous LED, time spent in the central arm, latency to head extension). Non-significant model terms, including interactions, were removed stepwise. The time spent in the central arm, the time taken to reach the LED and the latency to head retraction were truncated at 5 min. To ensure that this did not affect the results, we compared the regression with a truncated regression using the package ‘truncreg’ (https://cran.r-project.org/web/packages/truncreg/index.html). In no case did the truncated regression alter our findings. All analyses were performed using a linear scale of light intensities because of the narrow range used in the experiments. We compared our analyses using a linear scale with similar analyses using a log_10_ scale. In no case did the use of a log_10_ scale for light intensities alter the results. Pairwise differences between the control (dark Y-maze) and the treatment (illuminated Y-maze) were assessed using Wilcoxon rank sum tests from the ‘stats’ package. Significant model terms were assessed using Wald Chi-square tests (Type II ANOVA) from the ‘Car’ package (Fox and Weisberg, 2019) for the logistic regression, and *t*-test for linear models. Where appropriate, *P*-values were adjusted for multiple comparisons using false discovery rate correction (Benjamini and Hochberg, 1995).

## RESULTS

### The number of male glow-worms reaching a luminous green LED diminishes with time in a dark **Y**-maze

**Fig. 2.**
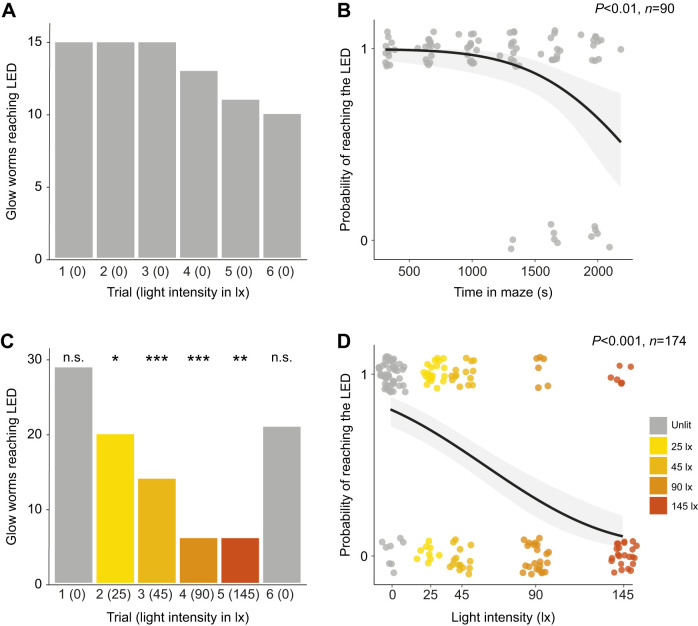
**Illumination impedes the ability of males to locate females in a Y-maze.** (A) The number of males reaching the luminous green LED across six successive trials in the unlit Y-maze. (B) A logistic regression (black line) predicting the probability of males reaching the LED given the total time spent by males in the unlit Y-maze. Jitter has been added to the probability values presented here and in D to permit visualisation of the data points. (C) As in A but in the presence of white light of differing intensities in the illuminated Y-maze. Significance levels correspond to the significance of the *G*-test comparing a trial in the lit maze with the equivalent trial in the unlit Y-maze, see Table 1. n.s., non-significant; *P<0.05; **P<0.01; ***P<0.001. (D) A logistic regression predicting the probability of males reaching the LED given the intensity of white illumination.

Male glow-worms were placed in the central arm of a dark Y-maze so that both distal ends of the choice arms were visible (Fig. 1). A green LED that mimicked the female glow (Fig. S1A) was turned on at the distal end of one of the arms, selected randomly. In response, all males (100%, *N*=44) walked towards and eventually reached the region within 1 cm of the distal end of the choice arm adjacent to the LED (Fig. 2A). Males were subjected to a further five consecutive trials (see Materials and Methods). The number of males reaching the LED at the end of each trial depended upon the total time across trials spent in the Y-maze (logistic regression: *N*=15, *n*=90, *Z*=3.013, d.f.=88, *P*<0.01; Fig. 2B); fewer males reached the luminous LED as the trials progressed, dropping to 67% (*N*=10) in the sixth trial (Fig. 2A). Of those males failing to reach the LED (*N*=5, *n*=11), some did not leave the central arm (*n*=5) or walked less than 50% of the length of a choice arm (*n*=2), whereas others walked towards the unlit LED (*n*=2) or stopped before reaching the luminous LED (*n*=2). Thus, though males detect and reach the luminous LED in an otherwise dark Y-maze, fewer males do so in later trials.

**Table 1.**
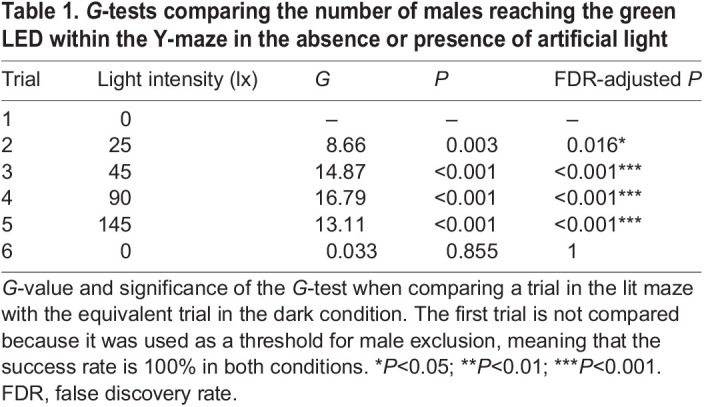
*G*-tests comparing the number of males reaching the green LED within the Y-maze in the absence or presence of artificial light

### Higher intensities of white illumination reduce the numbers of males reaching the luminous LED

We reproduced the effect of ALAN upon the ability of males to reach glowing females by diffusely illuminating the Y-maze with white light (Fig. S1B). As before, males experienced six successive trials. During the first trial, the Y-maze was dark other than the luminous green LED, the intensity of the white illumination increasing in each of the next four successive trials (trials 2–5). Males then experienced a final trial in which the maze was once again dark except for the LED. On each trial, a green LED was turned on at the distal end of one of the arms. At lower intensities (25 and 45 lx) of diffuse white illumination, approximately 70% (*N*=20) and 50% (*N*=14) of males reached the LED, respectively (Fig. 2C). At higher intensities (90 and 145 lx) on the fourth and fifth trials this dropped to just 21% (*N*=6; Fig. 2C). The number of males reaching the luminous LED on each trial depended significantly upon the intensity of the illumination (logistic regression: *N*=29, *n*=174, *Z*=6.045, d.f.=172, *P*<0.001; Fig. 2D). Of those males failing to reach the LED, there was no significant difference between the number that did not leave the central arm in which they were placed and those that chose the arm containing the unlit LED. In all four trials with white illumination (trials 2–5), significantly fewer males reached the luminous LED than on the equivalent trials in the dark Y-maze (Table 1). When returned to darkness in the sixth trial, there was no significant difference between those males that had experienced four previous trials with white illumination and those that experienced a dark Y-maze in which only the luminous LED was visible throughout (Table 1). Thus, whilst increasing intensities of white illumination prevents increasing numbers of males from reaching the luminous green LED, recovery is rapid after the illumination ceases.

### The time taken for males to reach the luminous LED is affected by the intensity of white illumination

**Fig. 3.**
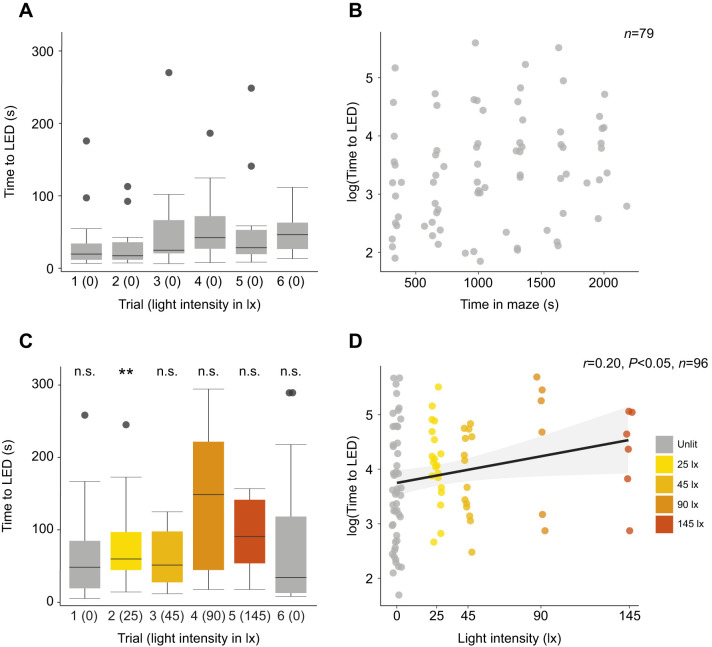
**Illumination increases the time taken by males to reach the luminous LED.** (A) The time taken [median±interquartile range (IQR)] by males to reach the LED in the unlit Y-maze across the six trials. (B) The total time spent in the unlit Y-maze versus the time taken by males to reach the LED. (C) As in A but in the presence of white light of differing intensities in the illuminated Y-maze. Significance levels correspond to the significance of the Wilcoxon test comparing a trial in the lit maze with the equivalent trial in the dark condition, see Table 2. n.s., non-significant; **P<0.01. (D) A linear model (black line) of the relationship between the time taken to reach the LED and the intensity of white light in the illuminated Y-maze.

To determine whether the white illumination affected the behaviour of those males that did reach the luminous green LED, we measured the time they took in each trial. Males in the dark Y-maze took 27.8 s (14.5–48.4 s) to reach the luminous LED (Fig. 3A). There was no significant relationship between the time taken for males to reach the luminous LED and the total time across trials spent in the dark Y-maze (linear model: *N*=15, *n*=79, *t*=1.897, d.f.=77, *P* =0.062; Fig. 3B). In the illuminated Y-maze, males took 48.25 s (19.3–84.7 s) to reach the luminous LED in the first (dark) trial, but in the second trial in the presence of the lowest intensity of white illumination this increased to 59.8 s (44.54–96.89 s) (Fig. 3C). Indeed, as the intensity of the white illumination increased, so too did the time taken by males to reach the luminous LED (linear model: *N*=29, *n*=96, t=2.186, d.f.=94, *P*=0.031; Fig. 3D). There was a significant difference in the time taken to reach the luminous LED between males in the illuminated and the dark Y-maze in the second but not in other trials (Table 2, Fig. 3). The time spent by glow-worm males in the central arm did not differ significantly in the sixth trial between the illuminated and dark Y-mazes (Table 2).

**Table 2.**
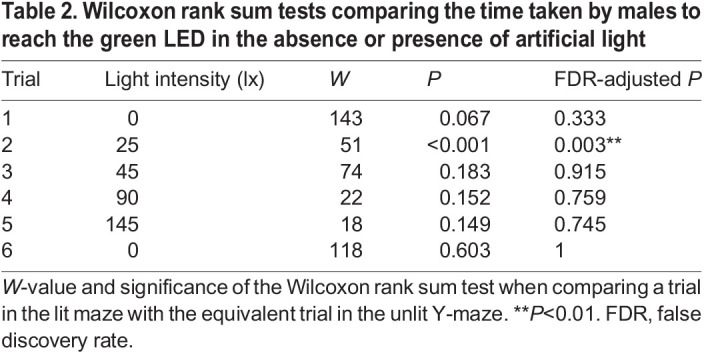
Wilcoxon rank sum tests comparing the time taken by males to reach the green LED in the absence or presence of artificial light

### The time spent by males in the central arm of the **Y**-maze increases with the intensity of white illumination

**Fig. 4.**
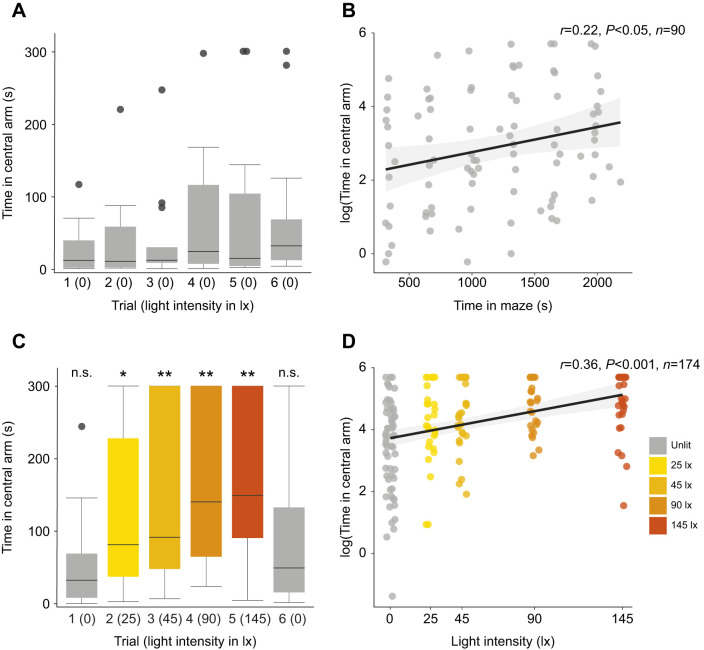
**Illumination increases the time spent by males in the central arm of the Y-maze.** (A) The time spent (median±IQR) by males in the central arm of the unlit Y-maze across the six trials. (B) A linear model (black line) of the relationship between the total time across all trials spent in the unlit Y-maze and the time spent in the central arm. (C) As in A but in the presence of white illumination of differing intensities. Significance levels correspond to the significance of the Wilcoxon test comparing a trial in the lit maze with the equivalent trial in the dark condition, see Table 3. n.s., non-significant; *P<0.05; **P<0.01. (D) A linear model (black line) of the relationship between the total time across all trials spent in the illuminated Y-maze and the intensity of white illumination.

We measured the timing of two other aspects of male movements within both the dark and illuminated Y-mazes: (i) the time males spent within the central arm in each trial; and (ii) the time males spend in close proximity to the luminous LED after reaching it. In the first trial, males in the dark Y-maze spent 12.2 s (2.2–39.9 s) in the central arm, but in the sixth trial this had increased to 32.4 s (12.7–68.7 s) (Fig. 4A). There was a significant increase in the time males spent in the central arm as the total time spent in the dark Y-maze across trials increased (linear model: *N*=15, *n*=90, *t*=2.379, d.f.=88, *P*<0.05; Fig. 4B). In the illuminated Y-maze, males spent 32.3 s (8.1–67 s) in the central arm in the first trial in which the only source of light was the luminous green LED, but in the second trial in the presence of the lowest intensity of white illumination this increased to 81.3 s (37.2–227.9 s) (Fig. 4C). Indeed, the intensity of the white illumination increased the time spent by males in the central arm (linear model: *N*=29, *n*=174, *t*=5.232, d.f.=172, *P*<0.001; Fig. 4D). The time spent in the central arm was significantly greater for males in all illuminated trials than the dark Y-maze (Table 3, Fig. 4). The time spent by glow-worm males in the central arm did not differ significantly in the sixth trial between the illuminated and dark Y-mazes (Table 3).

**Table 3.**
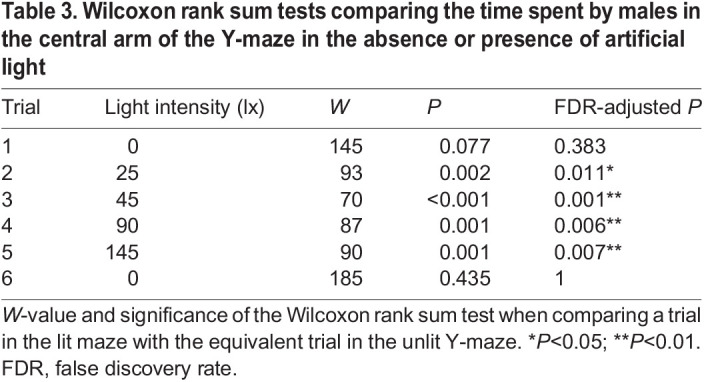
Wilcoxon rank sum tests comparing the time spent by males in the central arm of the Y-maze in the absence or presence of artificial light

In the dark Y-maze, males spent 174.85 s (82–236 s) in close proximity to the luminous LED within the distal region delineated by the threshold line during trials 2–5 (Fig. 1). There was no significant relationship between the time males spent in the vicinity of the luminous LED and the time spent in the Y-maze (linear model: *N*=15, *n*=79, *t*=−1.853, d.f.=77, *P*=0.068). In the illuminated Y-maze, males spent 121.775 s (74–159 s) of trials 2–5 in the vicinity of the luminous LED. There was no significant relationship between the time spent in the vicinity of the luminous LED and the intensity of the white illumination (linear model: *N*=29, *n*=96, t=−1.8, d.f.=94, *P*=0.075). The time spent by glow-worm males in the vicinity of the LED did not differ between the illuminated and dark Y-mazes in the first (Wilcoxon rank sum test: *N*=44, W=293, *P*=0.063) or sixth (Wilcoxon rank sum test: *N*=44, *W*=121, *P*=0.519) trials.

### Increasing intensity of white illumination increases time taken for males to extend their head beyond their head shield

**Fig. 5.**
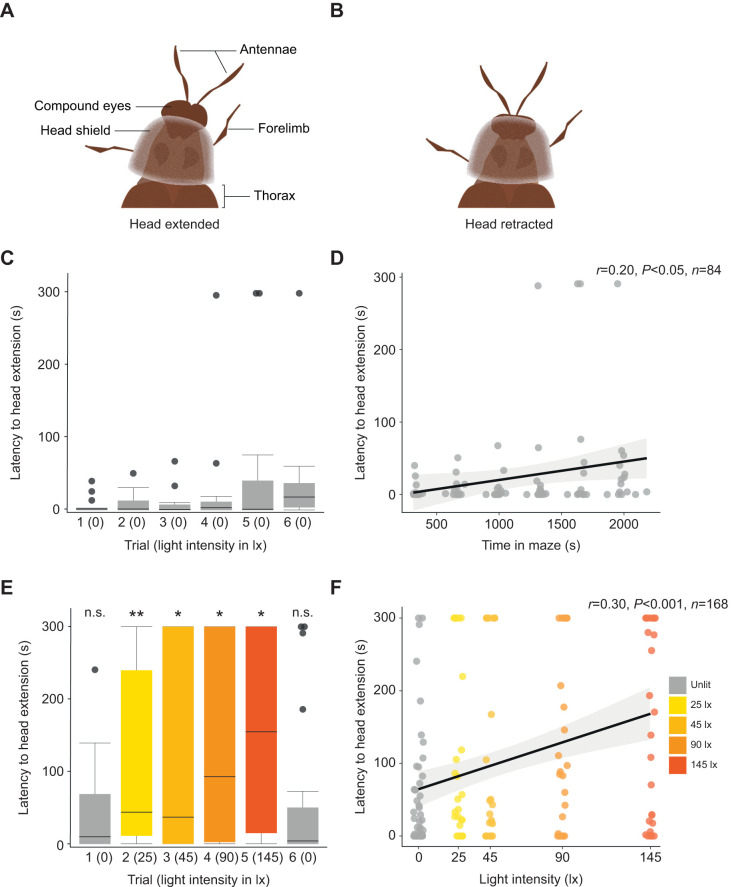
**Illumination increases the time spent by males with their head retracted beneath the head shield.** Schematic drawings of the anterior region of a male glow-worm with: (A) the head extended beyond and (B) the head retracted beneath the head shield. (C) The latency to male head extension in the unlit Y-maze across six successive trials. (D) A linear model of latency to head extension with the total time spent in the unlit Y-maze. (E) As in A but for the illuminated Y-maze. Significance levels correspond to the significance of the Wilcoxon test comparing a trial in the lit maze with the equivalent trial in the dark condition, see Table 4. n.s., non-significant; *P<0.05; **P<0.01. (F) A linear model of latency to head extension with the intensity of white light in the illuminated Y-maze.

Glow-worm males possess a cuticular shield that extends over the head, anteriorly from the pronotum, and may attenuate the light reaching the compound eyes (Tyler, 2002) (Fig. 5A,B). When males are actively searching for females, they typically extend their head beyond the head shield (Fig. 5A). We quantified the latency from the start of each trial to the male glow-worm extending their head beyond the head shield. in trials 2–5, males in the dark Y-maze spent 1.3 s (0–13.1 s) prior to extending their head beyond the head shield, just 0.4% of the 5 min trial (Fig. 5C). The latency to head extension increased significantly with the total time across trials spent in the dark Y-maze (linear model: *N*=15, *n*=84, *t*=2.353, d.f.=82, *P*<0.05; Fig. 5D). In the illuminated Y-maze, the latency for males to extend their head beyond the shield was 76.2 s (1.6–300 s) in trials 2–5, 25.4% of the 5 min trial (Fig. 5E). Males took significantly longer to extend their head in trials in which the Y-maze was illuminated in comparison with the dark Y-maze (Wilcoxon rank sum test, trials 2–5: *N*=42, *W*=1637, *P*<0.001; see Table 4 for pairwise tests). Indeed, in the dark Y-maze, just 14.3% of males kept their head retracted throughout the entire trial, but this increased to 55.6% of males in the illuminated Y-maze. There was no difference in the time males spent with their head retracted in the first or sixth trails when the Y-maze was dark except for the luminous LED (Table 4). Thus, male glow-worms spend more time with their head retracted under white illumination irrespective of the intensity (Fig. S3).

**Table 4.**
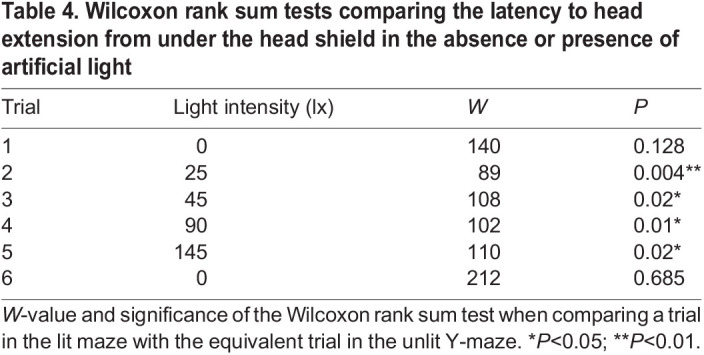
Wilcoxon rank sum tests comparing the latency to head extension from under the head shield in the absence or presence of artificial light

### The time taken by males to reach the luminous LED is influenced by the time spent in the central arm and the latency to head extension

**Fig. 6.**
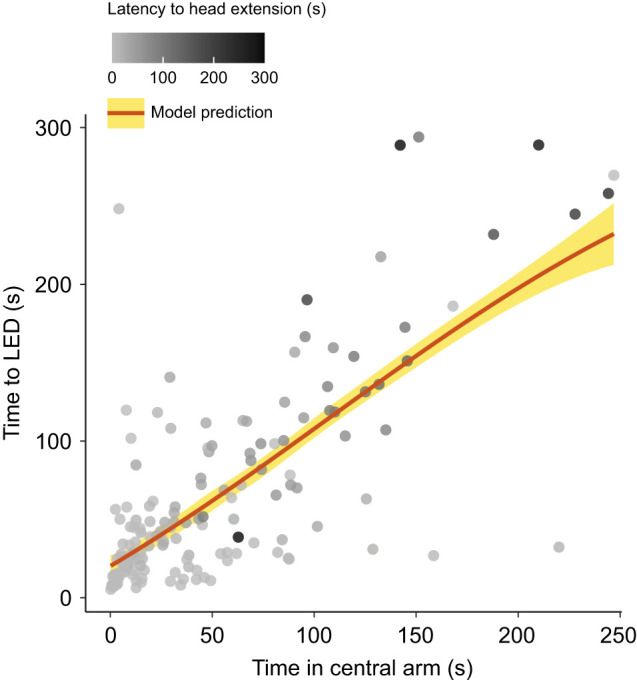
**The time taken to reach the luminous LED depends upon time in the central arm and latency to head extension.** A generalised linear model of the time taken by males to reach the luminous LED is predicted from two factors: the time spent by males in the central arm and their latency to head extension. The shading of individual points indicates the latency to head extension beyond the head shield. The red line indicates the model prediction and the yellow region shows the 95% confidence interval. For significance of the individual model terms, see Table S1.

To determine whether the time taken by males to reach the luminous LED was dependent upon other aspects of male glow-worm behaviour, we constructed a statistical model that incorporated both the time spent by males in the central arm of the Y-maze and the latency to extension of the males' head beyond the head shield. The model incorporating these behavioural factors was significant (Fig. 6A,B). Both the time spent by males in the central arm of the Y-maze and the latency to extension of the males' head beyond the head shield were significant predictors of the time taken by males to reach the luminous LED (GLM: *N*=37, *n*=165, t=4.99, *P*<0.001; Table S1). There was no significant interaction between the behavioural factors in the model.

## DISCUSSION

Our aim was to assess the behavioural level effects of artificial illumination on the ability of male glow-worms to reach females via their glow. To this end, we used a small Y-maze in which male glow-worm behaviour could be recorded throughout each trial, an approach that allowed us to monitor both their choices and the timing of their movements. All males could select the arm containing the dummy female (a luminous green LED) when no other light source illuminated the Y-maze. Artificial lighting affected both male glow-worms' choices and the timing of their movements; even the lowest light intensity reduced the percentage of males choosing the correct arm of the Y-maze to 75%, doubled the time taken by males to reach the luminous LED, more than doubled the time males spent in the central arm during a trial, and produced a 4.5-fold increase in the latency of males’ head extension beyond their head shield. These effects became more pronounced as the light intensity increased. Together, our results show deleterious effects of white illumination on the ability of male glow-worms to reach the glow emitted by females at the behavioural level that have not been demonstrated in previous ecological experiments.

### Decline in male glow-worm mate choice

Male glow-worms experienced six sequential trials in the Y-maze separated only by the time taken to reposition the males in the central arm. In the absence of the white illumination, the number of males reaching the dummy female dropped from 100% in the first trial to ∼67% in the sixth trial. Most males (82%) that did not choose the dummy female remained near the central arm, the remainder (18%) choosing the other arm of the Y-maze. This decline in males reaching the dummy female cannot be attributed to sensory detection, given that external conditions were the same in each trial. It is also unlikely to be a consequence of a reduction in resources needed for muscle activity because adult glow-worms do not eat (Tyler, 2002). There are numerous possible explanations, such as habituation owing to repeated exposure to the same stimulus or a central process that reduces males' motivation to walk towards the glow despite the position changing on each trial. Irrespective of the mechanism involved, the decline in male attraction to the female glow undermines the view of this behaviour as being highly stereotyped. One (of many) adaptive explanations is that repeated unsuccessful trials within the Y-maze reproduce a scenario in which males visit multiple females on a single evening but discover that they are already mating. Indeed, females do not stop glowing even after mating has begun (Tyler 2002), thereby continuing to attract males. Rather than expending resources continuing to search for potential mates, males may wait in the vicinity where there is a higher probability of new females emerging on subsequent evenings.

### Relevance of lighting

The interpretation of experiments that seek to understand the impact of artificial lighting on behavioural processes depends upon the emission spectra of the light sources and their intensity. We quantified light intensity in lux. Although the spectral sensitivity for *L. noctiluca* photoreceptors has not been determined to our knowledge, two lines of evidence suggest that lux is an appropriate measure of apparent brightness of the LED and broad-spectrum light because: (1) spectral sensitivities for other lampyrids indicate at least one class of photoreceptors with a maximal spectral sensitivity of 500–560 nm (reviewed in van der Kooi et al., 2021), close to that of the standard luminosity function (Stiles and Burch, 1955); and (2) the broad emission spectrum of the female glow has a ∼546 nm peak (Booth et al., 2004; De Cock, 2004). Evidence from other lampyrid beetles (Lall, 1981) suggests that the glow is likely to be a signal that male *L. noctiluca* photoreceptors are spectrally tuned to detect. To our knowledge, other lampyrid photoreceptor classes have not been determined (reviewed in van der Kooi et al., 2021). Thus, measurements using lux are an appropriate approximation for the apparent brightness of our light sources to *L. noctiluca* males.

To mimic the glow of a female glow-worm, we used a green 555 nm LED with an emission spectrum (Fig. S1) that closely resembles that of females, which produce a glow with a peak at ∼546 nm (Booth et al., 2004; De Cock, 2004). Similar LEDs mounted on small funnel traps are capable of attracting large numbers of males in field experiments (e.g. Ineichen and Rüttimann, 2012; Bird and Parker, 2014; Stewart et al., 2020; Van den Broeck et al., 2021; Kivelä et al., 2023), suggesting that they are an appropriate substitute for the glow of females. In the Y-maze used here, male glow-worms were all able to reach the dummy female when no other lighting source was present. This may be a consequence of the intensity of the green LED, which was 1 lx at the point where glow-worms were placed into the central arm. This intensity is likely greater than that of the female glow when detected by males, which may be further away and viewing the glow through undergrowth. In the field, males likely experience higher intensities after landing and beginning to walk towards the glowing female (J.E.N. and A.J.A.S., unpublished observation). The behaviour we observed within the Y-maze most likely represents the walking of male glow-worms as they search for females after landing.

We used a diffuse ‘warm’ white light to reproduce the effect of ALAN on the ability of male glow-worms to locate females (Fig. S1B). This white light had a broad emission spectrum with a peak at ∼614 nm but lacked the pronounced short wavelength peak that is present in many white streetlights (Elvidge et al., 2010; Rowse et al., 2016). The absence of the short wavelength peak, whilst not completely abolishing short wavelengths, reduced the potential for these wavelengths to interfere with the attraction of males to the glow produced by green LEDs used to mimic female glow-worms (Booth et al., 2004). The presence of short wavelengths may have confounded interpretation of the general effects of light intensity with specific effects of short wavelengths. The pronounced effects of white illumination in our experiments suggests that light in the 500–700 nm range may contribute to the deleterious effect of ALAN on glow-worms. The presence of short (blue) wavelengths found in modern streetlights may exacerbate the inhibitory effect of white illumination on male glow-worm behaviour.

The Y-maze was illuminated with a range of white light intensities from 25 to 145 lx at 30 cm from the light source, which correspond to similar or slightly higher light intensities than those found in other ecological studies. For example, Stewart et al. (2020) used light levels that ranged from 375 lx at 1 m from the light source to ∼0.12 lx at 55 m from the light source in field experiments that exposed dummy females to ALAN to measure the effects on male attraction. Similar recent field studies of *L. noctiluca* have used light intensities corresponding to the middle range of those measured by Stewart et al. (2020). For example, Elgert et al. (2020) and Kivelä et al. (2023) used between 5 and 8 lx to illuminate dummy females and assess the effects on male attraction. However, studies of other species have used higher light intensities, such as Firebaugh and Haynes (2016), who used 300 lx when studying the effects of artificial lighting on courtship of the crepuscular firefly *Photinus pyralis*, and Owens and Lewis (2022), who used up to 240 lx in a laboratory study of courtship of the semi-nocturnal firefly *Photinus obscurellus*. Nevertheless, the light intensities used in this study should be considered at the higher end of those that could be experienced by *L. noctiluca*, which are nocturnal. Yet the potential effects of the higher light intensities used in this study are likely offset by the high intensity of the dummy female green LED (1 lx) relative to the intensity of the female glow. This suggests that the effects of artificial illumination we document here are relevant to field conditions in which the female glow and ALAN may both be dimmer.

### Comparison with field studies

The majority of studies examining the effect of artificial lighting on the ability of males to reach females (or green LEDs acting as dummy females) have been executed in the field (e.g. Ineichen and Rüttimann, 2012; Bird and Parker, 2014; Stewart et al., 2020; Van den Broeck et al., 2021; Kivelä et al., 2023). Each of these studies shows that artificial lighting impairs the ability of males to reach females, though how it does so remains unclear. One likely scenario is that artificial lighting prevents flying males that are searching the environment from detecting the female glow over long distances (possibly >1 m) so that they seek alternative nearby females not exposed to artificial lighting with which to mate. If this is correct, the presence of alternative females in the local environment may contribute to the severity of the effect of artificial lighting on the numbers of males reaching females. However, the current inability to measure the distances over which males can detect females limits inferences about the point at which artificial lighting interferes with ability of males to locate females.

Our Y-maze results demonstrate that white illumination can have marked effects on the ability of males to reach females even over short distances whilst walking. Although males are usually assumed to detect females whilst flying (Tyler, 2002), many do not land directly on the female but rather land on nearby vegetation and walk to reach the female (J.E.N. and A.J.A.S., unpublished observation). We found a significant reduction in the number of males reaching females with increasing intensity of white illumination; from just 10 cm away, only 70% of males were able to locate a 1 lx female mimicking LED in the presence of 25 lx artificial lighting, dropping to 50% at 45 lx. At higher light intensities, fewer than 25% of males were able to reach the LED. Broadly, these results agree with ecological experiments that show that even dim white illumination can reduce the numbers of males reaching dummy females.

### Behavioural effects of white illumination

Our approach allows us to go beyond the numbers of males arriving at a luminous LED to consider behavioural level effects of white illumination. We found that white illumination increased: (i) the time taken by males to reach the luminous LED; (ii) the time spent by males in the central arm; and (iii) the latency of males' head extension beyond their head shield. The increase in the time taken for males to reach females is predicted by the time spent in the central arm of the Y-maze and the latency of head extension beyond the head shield. Both of these behaviours show male glow-worms avoiding exposure to the white illumination, suggesting that this stimulus is aversive and strongly inhibits males from searching for females. This conclusion is supported by observations of male glow-worms seeking out shade at the base of the central arm, thereby reducing their exposure to the white illumination. The increased time taken for males to reach females represents a cost to males that may translate into lost opportunities to reproduce given that males have just a few hours in which to detect and reach females each evening (J.E.N. and A.J.A.S., unpublished observation). Such behavioural costs are not detected by ecological experiments that measure the numbers of males caught in female-mimicking traps. Moreover, it raises the possibility that males entering regions brightly lit by ALAN may become trapped and unable to escape for long periods, demonstrating that there are several means by which artificial lighting can impair the ability of males to reach female glow-worms in a natural setting.

### Is recovery after artificial lighting is removed related to retraction of the head beneath the head shield?

Following exposure to the highest intensity of white light, males experienced a final trial in which the Y-maze was returned to darkness with only one green LED lit. The number of males locating the green LED increased and the time taken reduced to similar levels to those that had not been exposed to white light. One explanation is that males could avoid or mitigate their exposure to high light intensities. Male glow-worms possess a head shield that extends anteriorly from the pronotum (Tyler, 2002). When the head is retracted, the head shield covers the compound eyes, potentially attenuating the intensity of light they receive, though it may also act as a selective filter attenuating some wavelengths more than others. Potentially, this may be a strategy similar to blinking in some vertebrates or wearing sunglasses for humans. Male glow-worms searching for females at night typically extend the head beyond the head shield (E.M.M., A.S.D.F. and J.E.N., unpublished observation). In the dark Y-maze, males typically had their head extended beyond the shield for the majority of each trial. Conversely, when they were exposed to white light they kept their head retracted beneath the shield for the majority of each trial. The retraction of the head beneath the shield may reduce the impact of the white light in the trials, particularly at the lowest light intensity. Nevertheless, head retraction may reduce the ability of males to detect and reach females in cases where they are navigating through vegetation in which the female may not be on the same horizontal plane, as the luminous LED was in our experiments. If head retraction beneath the shield does attenuate the light intensity reaching the photoreceptors of the compound eye, this may explain in part the rapid recovery of male glow-worms in the final dark trial.

## Supplementary Material

10.1242/jexbio.245760_sup1 Supplementary information
