# Supplementary material for: Artificial light impairs local attraction to females in male glow-worms
Source: J Exp Biol. 2023 Jun 14;226(11):jeb245760. doi: 10.1242/jeb.245760 (PMC10281516; doi:10.1242/jeb.245760)
Supplement: Supplementary information [file jexbio-226-245760-s1.pdf]

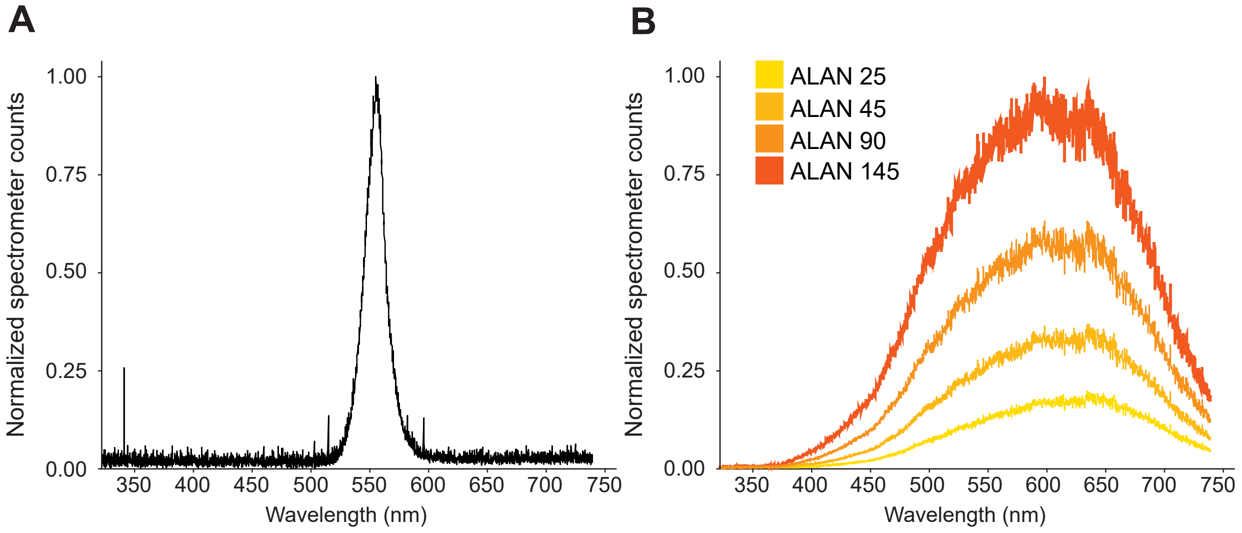

Fig. S1. The emission spectra of the light sources used in the Y-maze. A. The emission spectrum of the green LED used to mimic the glow of female glow-worms. The narrow spikes on the spectrum are a consequence of dark/instrument noise, which was not removed. **B.** The emission spectrum of the warm 'white' light used to illuminate the Y-maze.

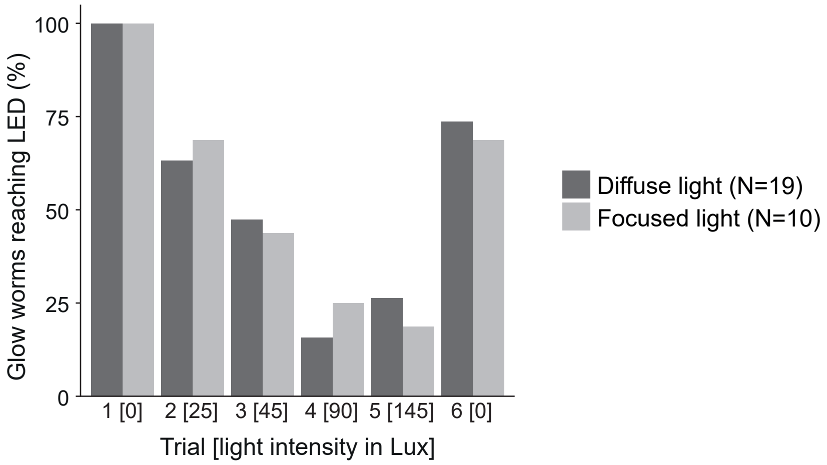

Fig. S2. Restricting white illumination to a local region at the distal end of arm Y-maze arm did not alter the percentage of male glow worms reaching the green LED. A comparison of the percentage of male-glow worms that reach the LED in the diffuse white illumination regime (dark grey) and when white illumination is focussed on a local region at the distal end of each arm of the Y-maze (light grey). There is no statistical difference between the two lighting conditions (not shown).

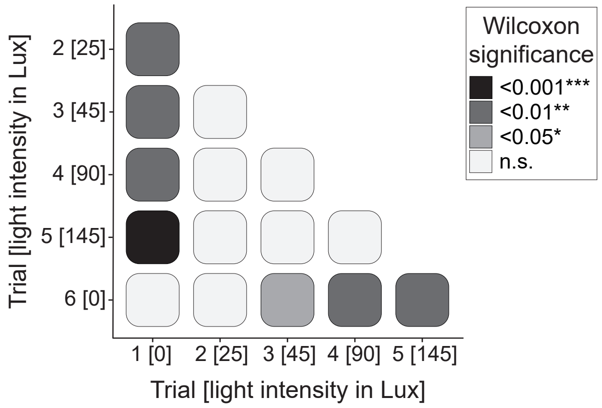

Fig. S3. Males spend more time with their head retracted in the presence of artificial light regardless of light intensity. Significance of the Wilcoxon Rank Sum when comparing each pair of trials in the illuminated Y-maze.

Journal of Experimental Biology • Supplementary information

Table S1. The time taken by males to reach the green LED is determined by the time spent in the central arm and the latency to head extraction in all conditions. Linear model with the time taken to reach the green LED as the response variable and the time spent in the central arm and latency to head extension as independent predictors. Significant of model terms were assessed using T-test.

No. males	Predictor	n	Estimate	t-value	p-value
37	Intercept	165	20.7	4.99	<0.001
	Time in central arm		0.61	8.03	<0.001
	Latency to head extension		0.42	5.84	<0.001

Dataset 1

Click here to download Dataset 1

Journal of Experimental Biology • Supplementary information
